# Feasibility Study for Wearable Sensor-Based Vibrotactile Feedback for Posture and Muscle Activation in a Relevant Dentistry Setting

**DOI:** 10.3390/s25185891

**Published:** 2025-09-20

**Authors:** Demir Tuken, Samuel J. Murphy, Robert D. Bowers, Rachel V. Vitali

**Affiliations:** 1 Department of Biomedical Engineering, University of Iowa, Iowa City, IA 52242, USA; demir-tuken@uiowa.edu; 2 Department of Mechanical Engineering, University of Iowa, 103 S. Capitol St., 2136 Seamans Center, Iowa City, IA 52242, USA; samuel-murphy@uiowa.edu; 3 Department of Family Dentistry, University of Iowa, Iowa City, IA 52242, USA; robert-bowers@uiowa.edu

**Keywords:** wearable sensors, inertial measurement unit, posture, spinal orientation, surface electromyography, muscle recruitment, haptic feedback, ergonomics, musculoskeletal disorders

## Abstract

This feasibility study evaluated a wearable sensor-based haptic feedback system designed to promote ergonomic awareness and influence posture and muscle activation patterns during a standard dental procedure. Inertial measurement units (IMUs) monitored posture by tracking back and neck angles, while four surface electromyography sensors recorded muscle activation in the lower erector spinae (LES) and upper trapezius (UT) muscles. Two IMUs with vibrotactile motors delivered real-time haptic feedback when participants maintained mechanically disadvantageous postures for extended periods during a cast metal crown preparation procedure on a manikin typodont. Data from four dental students participating in a total of 24 trials, half with and half without feedback, were analyzed via a two-way ANOVA to determine the effects of feedback and activity (e.g., inspections or drilling) on posture and muscle activation. Feedback slightly increased neck angles, but back angles remained nominally unchanged. Reduced UT activation and increased right LES activation suggests altered muscle recruitment strategies. Heatmap and RULA analyses indicated a shift toward more varied and potentially safer postural distributions during feedback trials. Postural and muscle activation data were also analyzed across four activity labels, which revealed that Drilling was consistently associated with higher ergonomic risk. Real-time haptic feedback influenced posture and muscle activation in dental students, particularly by reducing UT strain despite increased neck flexion. These findings support the integration of wearable feedback systems into preclinical training to enhance ergonomic awareness and potentially reduce the risk of developing musculoskeletal disorders, to which dentists are particularly prone.

## 1. Introduction

Dental professionals perform lengthy procedures that often require them to assume mechanically disadvantageous postures [1]. Consequently, musculoskeletal disorders (MSDs) have been widely reported in dental professionals, ranging globally from 10.8% to 92.9% [2,3]. According to a 2018 systematic literature review and meta-analysis, MSDs were most reported in the neck (58.5%), lower back (56.4%), shoulder (43.1%), and upper back (41.1%) [4]. Since MSDs often develop gradually over years or even decades, isolating a single causative factor is inherently challenging, particularly when accounting for the cumulative effects of dental education and subsequent clinical practice [5]. Nonetheless, prior research has identified correlations between perceived pain and the adoption of mechanically disadvantageous postures in dentistry, suggesting that such postures may serve as early risk indicators for developing MSDs [6].

According to a 2020 systematic review, there is some evidence suggesting that ergonomic interventions including magnification loupes/microscopes, prismatic spectacles, training courses, specially designed dental chairs, and instruments have the potential to reduce the severity of MSDs by improving work posture among dental professionals [3]. The extent to which ergonomic interventions are applied in dentistry, as well as the specific barriers to their implementation, remains poorly understood. Strengthening the body of high-quality, objective evidence demonstrating the effectiveness of different interventions will be essential for identifying the best strategies to prioritize incorporating into dental education and clinical practice [1].

Ergonomics education can be taught through awareness and training exercises. Ergonomic awareness refers to an individual’s general understanding of ergonomic principles, whereas ergonomic training involves structured instruction and practice aimed at applying those principles effectively in specific tasks or work environments. The Commission on Dental Accreditation (CODA), which oversees accreditation for U.S. dental schools, does not currently mandate specific ergonomic training as part of its core standard for predoctoral education, leaving ergonomics training and awareness as a voluntary pursuit in dental education [7]. The absence of ergonomic training has been found to exacerbate the effects of developing MSDs in dental school, which is direct evidence that ergonomics awareness alone is not a solution to reducing the risk of developing MSDs in new dentists [8]. Awareness of suggested ergonomic practices alone has been shown to be insufficient to combat the rising prevalence of MSDs within dentistry, though ergonomic training can reduce reported pain [9]. In a 2016 study, interventions targeting muscles for posture and balance were assessed six months post-intervention [10]. The intervention group engaged in an eight-week ergonomic intervention that included ergonomic awareness lectures, workstation modification, ergonomic training, surveying ergonomics at the workstation, and a regular exercise program. While the authors reported statistically significant differences in MSDs in multiple body parts [10], a systematic review determined that the study provided very low-quality evidence for several different reasons, including “inappropriate use of statistical tests and calculation” ([1] p. 19).

Incorporating ergonomics into dental education is complicated by the tendency of dental students to sacrifice healthy ergonomic behaviors early on in their clinical training. This situation often occurs in favor of completing clinical procedures more efficiently, particularly when indirect vision through mirrors is challenging or when suctioning must be performed without the assistance of a dedicated dental assistant. Additionally, students are unlikely to experience significant pain or discomfort during the early stages of their education, yet the repetitive nature of clinical tasks and the development of muscle memory can lead to the onset of MSDs over time. Therefore, continuous ergonomic training, ideally introduced concurrently with instruction on technical procedures, is essential to effectively address the high rates of MSDs among dental professionals.

One promising approach to training dental students to adopt healthy biomechanical behaviors is through real-time feedback provided by wearable sensors, whose utility has been demonstrated in multiple settings [11,12,13]. Wearable sensor-driven feedback systems alert users when they maintain mechanically disadvantageous postures for a prolonged period of time, ideally training them to become more mindful of their biomechanical behaviors during typical clinical procedures. As an alternative to previous work that has relied on human observers for real-time feedback, wearable technology offers a scalable, personalized, and calibrated solution or adjunctive tool to support ergonomics training. The goal is not to eliminate specific postures entirely but to foster an environment that prompts frequent postural adjustments throughout a procedure. This approach is guided by the success of intraoperative “micro-breaks” implemented in other surgical settings [14,15], which have been shown to reduce the duration of static and often asymmetric loading of their muscles and joints [16]. Furthermore, these posture changes promote the recruitment of a more diverse set of muscles groups, allowing for intermittent recovery during procedures.

Wearable sensors like inertial measurement units (IMUs) have been utilized extensively for monitoring posture. For example, Porta et al. [17] used a single IMU to monitor trunk flexion in nurses to quantify how much time was spent in potentially harmful postures. The authors determined that the IMU-based approach was a promising alternative for capturing different biomechanical behaviors over long periods of time compared to traditional methods requiring a human observer. Several studies explicitly integrated the rapid upper limb assessment (RULA) [18] in combination with IMU data to evaluate the ergonomic risks associated with different postures. For example, Tanthuwapathom et al. [19] and Humadi et al. [20] both reported strong agreement between IMU-based and traditional RULA assessments, supporting the use of IMUs for ergonomic screening. However, Nowara et al. [21] found that applying RULA using IMU data systematically produced higher risk scores than applying RULA using traditional methods, particularly for the arm and wrist, in a study population of 130 dentists and dental assistants. Finally, Carbonaro et al. [22] monitored one surgeon’s posture during laproscopic surgery to identify prolonged periods of neck extension and trunk flexion. Their results suggested that the surgeon’s RULA scores were high enough to warrant a change in operating conditions. Importantly, the authors also acknowledged the value of also monitoring muscle activation via electromyography (EMG). These studies suggest that, while IMU-based RULA offers greater discriminatory power and objectivity, methodological differences must be considered when interpreting or comparing results across studies or with historical data. Further, there is an opportunity to enhance the ergonomic risk assessment with the addition of EMG sensors.

To define what constitutes a mechanically disadvantageous posture in dental settings, this study will also utilize the RULA for guidance [18]. As described above, this tool enables evaluators to identify a dentist’s worst-case posture, which can guide how to set thresholds for recognizing disadvantageous postures during procedures [23]. A significant limitation of RULA is its inability to accurately account for posture duration and muscle recovery time [24], both of which are of interest in this study. Thus, RULA is used in concordance with muscle activation measurements to better understand how the feedback influences not only their posture, but also muscle recruitment. Building upon prior research that has explored the relationship between real-time posture feedback and muscle recruitment [25], this feasibility study investigates the impact of a novel wearable sensor-based feedback system on postural and muscle activation behaviors during a typical dental procedure.

## 2. Materials and Methods

### 2.1. Participants

Fourth-year dental students at the University of Iowa were invited via email to voluntarily participate in the study. Their curriculum included several lectures on ergonomic awareness; however, it lacked structured ergonomic training exercises beyond general feedback provided during simulation and clinical sessions. The exclusion criteria included (1) muscle injuries, (2) recent fractures, (3) vestibular disorders, and (4) heart conditions, which were assessed through self-reporting via responses to the recruitment email and reaffirmed by the research team when reviewing the study’s informed consent. This study received approval from the Institutional Review Board (IRB) of the University of Iowa.

### 2.2. Experimental Procedure

**Figure 1:**
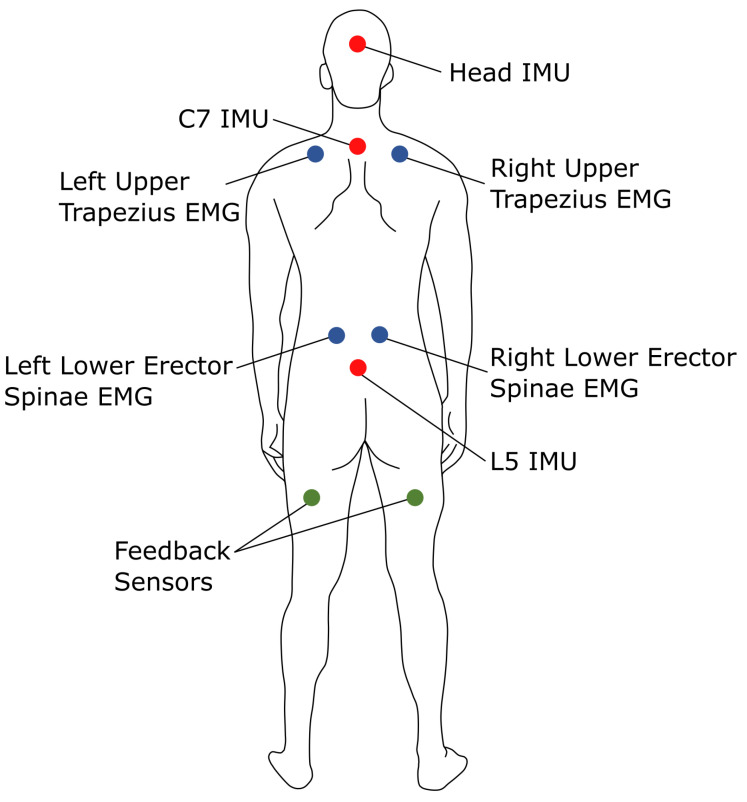
Placement of surface electromyography (sEMG) sensors (blue), inertial measurement units (IMUs; red), and haptic feedback devices (green).

Each of the four participants who volunteered for the study self-reported their height, weight, and sex. Muscle activation was recorded with surface electromyography (sEMG) sensors (Cometa, Bareggio, Italy) placed on the lower erector spinae (LES) and upper trapezius (UT) muscles, both of which are involved in posture-related musculoskeletal discomfort and ergonomic equipment [26,27,28,29]. Three inertial measurement units (IMUs; SageMotion, Kalispell, MT, USA) were taped to the L5, C7, and posterior head positions to measure the orientations of the pelvis, torso, and head, respectively. The IMUs also include vibrotactile motors for haptic feedback. Two IMUs were placed in the user’s pockets to deliver feedback via leg vibrations, mimicking a cell phone alert. One was placed in the participants’ right and left pocket to give neck and back angle threshold feedback. Figure 1 illustrates the sensor placement.

Each participant completed six cast metal crown preparation procedures on a manikin typodont in an enclosed operatory environment with typical armamentarium for crown preparation procedures. The participants were asked to complete at least three trials on molars of their choice, with the remaining on teeth of their choice. This experimental design was chosen to minimize repetition-related bias, as the dental students were still in the process of learning the procedure and perfecting their technique. All participants wore through the lens (TTL) dental loupes equipped with headlights. Using a randomized order, three trials were conducted with feedback and three trials were conducted without feedback. Two GoPro Hero 8 cameras were positioned at a roughly 60-degree angle relative to one another and pointed at the manikin to record the students’ hand movements and tool usage.

### 2.3. Surface Electromyography

The UT and LES muscles on both sides were located by anthropometric measurements and contractional palpation. The sEMG sensors have a differential design and are 1 × 2.5 × 0.5 cm. Kendall 530 foam electrodes (Balego, Minneapolis, MN, USA) were used to adhere the sensors to the participants’ skin after being cleaned with isopropyl alcohol. The sensors included two electrodes in a differential configuration, with each electrode measuring 1.5 inches in diameter.

Isometric maximum voluntary contractions (MVCs) were measured for each muscle via the SENIAM protocol [30]. After electrode placement, participants contracted their muscles three times at self-perceived levels of 20%, 50%, and 70% of their maximal effort. A protocol was followed to measure the MVC for each muscle. For the UT, participants progressed through increasing effort levels culminating in a maximal effort to complete a 35-pound dumbbell shrug with both muscles that was subsequently held for three seconds. Each exertion was followed by a 60 s rest period and repeated three times [31]. For the LES, participants followed a warm-up protocol prior to a maximal-effort 5 s superman exercise to activate lower trunk extension. This was also repeated three times with 1 min rest intervals between each exertion [32]. During each MVC trial, a research team member applied opposing force to the participant’s motion, and verbal encouragement was provided to help participants achieve peak muscle activation.

The sEMG sensors recorded data at 2000 Hz. Following a similar processing pipeline [33], the post-processing of the raw EMG data included a bandpass filter, full wave rectification, and low pass filter. The cutoff frequencies for the bandpass filter were 25 Hz and 480 Hz, and the cutoff frequency for the low-pass filter was 20 Hz. The frequency cutoffs were determined based on previous literature in muscle activity data acquisition in workplace settings [34]. The sEMG data were then normalized to the highest post-processed reading in the MVC test, yielding muscle activation percentages. The root mean square (RMS) for each trial was taken in non-enveloping 10-millisecond intervals [35]. Integrated EMG (iEMG) was calculated by summing these sEMG RMS intervals over time to quantify total muscle activation during the period (e.g., over a trial).

### 2.4. Inertial Measurement Units

The L5 and C7 vertebrates were located by palpitation. IMUs were then taped on the L5 and C7, and strapped to the posterior head; the IMUs were used to track neck and back angles. The design of the IMUs includes vibrotactile motors that can produce feedback at 300 Hz. Thus, two feedback sensors that were used for a neck (back) angle threshold were placed in the participants’ right (left) pocket. The participants stood in a neutral posture for 10 s for sensor calibration, and their neutral back and head angles were recorded. Following calibration, the 35-degree and 20-degree feedback threshold angles were calculated for both angles using each participant’s neutral posture. Neck flexion beyond 20 degrees and back flexion between 20–60 degrees are considered higher ergonomic risks under the RULA framework. However, preliminary testing showed that a 20-degree threshold for back flexion was too strict for the dental tasks involved, resulting in near-constant feedback. To improve practicality, the threshold was adjusted to 35 degrees. Feedback was sent to the user if they exceed a threshold for more than a minute, with the feedback ceasing once the user changed posture to be under the threshold angles. The one-minute threshold was chosen for several reasons. First, feedback was designed to avoid becoming a nuisance or disrupting the user, especially during fine motor tasks in dental procedures that demand sustained concentration. Additionally, the original RULA framework [18] highlights that static postures held for over a minute carry higher ergonomic risk. If the user changed postures to an angle under the threshold for more than 30 s after the initial feedback signal was sent, the one-minute counter reset. If the user reverted to a mechanically disadvantageous posture within 30 s of the first feedback signal, another feedback signal was sent to the user intended to encourage the user to genuinely adopt a different posture.

Each IMU contained a triaxial accelerometer, angular rate gyroscope, and magnetometer. The orientation is estimated via a proprietary algorithm (see [36] for further information regarding the algorithm and see [37,38] for examples of similarly validated orientation algorithms). The roll Euler angle was calculated with

(1) $$\theta =arctan\left(q_{0},q_{1}\right)-90{}^{\circ}$$

where $q_{0}$ and $q_{1}$ are quaternion elements, and subtracting 90° relates the sensor’s orientation to vertical. The posture analysis was conducted in real time using Python (version 3.12.1), while muscle activation analysis and all post-processing were performed in MATLAB (version 2024a).

### 2.5. Data Analysis

**Figure 2:**
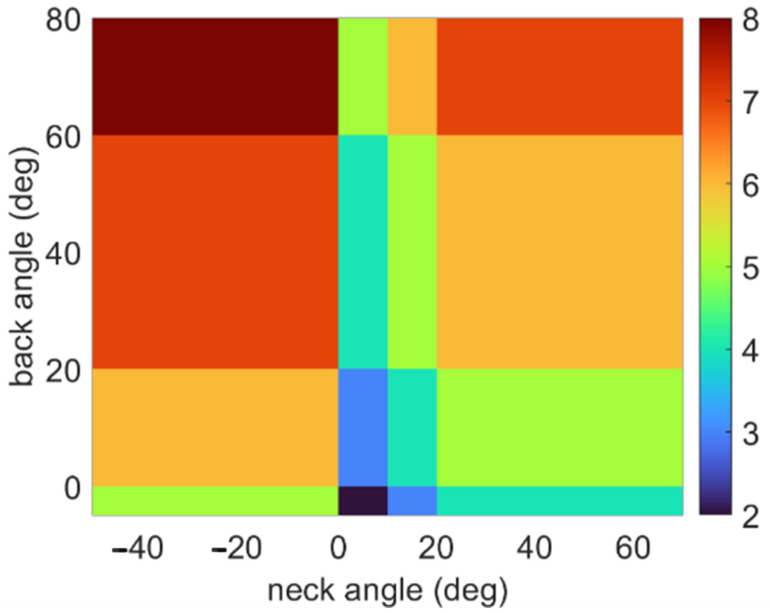
RULA scores for different combinations of back and neck angles. Lower scores (blue) denote safer (ergonomically advantageous) postures, whereas higher scores (red) denote more dangerous (ergonomically disadvantageous) postures.

One trial for Participant 1 incurred technical difficulties during a trial without feedback, and no data was collected. The sEMG sensors attached to the erector spinae muscles unknowingly detached halfway through the testing session for Participant 3, which resulted in there being no usable data for the feedback condition for these two muscles. The posture and muscle activation data is presented to show the ergonomic risk associated with different spinal orientations in Figure 2.

Video data were processed using MaxQDA software (version 24.9) with the following labels assigned: (1) Drilling, (2) Inspection, (3) Drill Bit Switch, and (4) Air/Water Application. These labels were used to characterize how the presence of feedback alters the participant’s biomechanical behaviors during the cast metal crown procedure. The average neck angle, back angle, and iEMG were taken for each labeled section.

Two-way analysis of variance (ANOVA) was conducted to examine the independent main effects of activity type and feedback on spinal orientations and integrated EMG values. Effect sizes ($\eta^{2}$) were calculated to quantify the magnitude of the effect that the factor had on the dependent variable [39]. Post hoc analyses via Tukey pairwise comparisons were conducted for ANOVAs with significant F-statistics. Effect sizes (Cohen’s *d*) were calculated to evaluate the magnitudes of the differences between groups [39]. All the statistical tests were conducted using a significance level of α=0.05.

For the ANOVA results, the F-statistics with their associated between-group and within-group degrees of freedom, *p*-values, and effect sizes ($\eta^{2}$) are reported. The F-statistic represents the ratio of the variance between group means and variance within each group, where a higher F-value suggests that the group means are more spread out than would be expected by chance. The *p*-values are considered statistically significant if they are below a significance level of α=0.05. The effect sizes ($\eta^{2}$) are calculated to quantify the magnitude of the main effect (activity or feedback) on the metric being tested. They are considered small, medium, or large at values of 0.01, 0.06, or 0.14, respectively [39].

For ANOVA tests with significant F-statistics (i.e., p<α=0.05), the post hoc Tukey analyses are reported for only statistically significant pairwise comparisons, specifically the *p*-values and effect sizes (Cohen’s *d*). The *p*-values are considered statistically significant if they are below the aforementioned significance level. The effect sizes are calculated to evaluate the magnitudes of the differences between activities or feedback conditions. They are considered small, medium, or large at values of 0.2, 0.5, or 0.8, respectively [39].

## 3. Results

The results are first reported by descriptive statistics for posture and muscle activation for each participant. Then, the ergonomic assessments via heatmaps and RULA analyses are offered. This section concludes with the statistical results.

### 3.1. Descriptive Statistics by Participant

The average amount of time per trial for each participant is presented in Table 1. Interestingly, three participants spent less time completing the procedure with feedback. The feedback enabled participants to slightly decrease their average neck and back angles. However, participant 3 increased their back angles to accommodate a decrease in neck angles while participant 4 slightly increased both angles in the presence of feedback.

**Figure 3:**
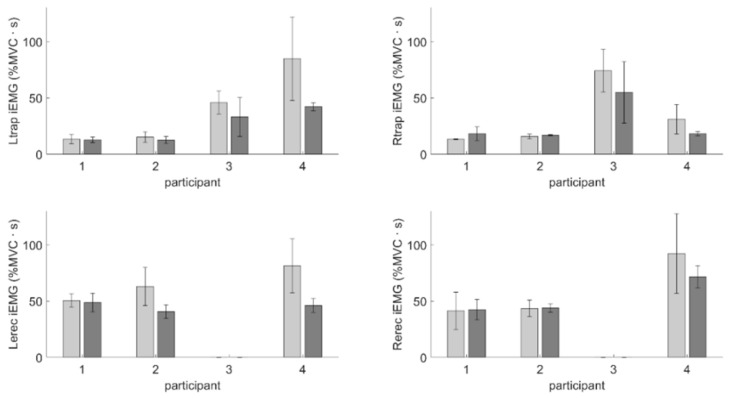
Average integrated EMG (iEMG) for upper trapezius (**top row**) and lower erector spinae (**bottom row**) with standard deviation bars for when feedback was present (dark gray) and was not present (light gray).

The average integrated EMG (iEMG) for each participant’s evaluated muscles is presented in Figure 3. On average, participants exhibited either nominally unchanged or decreased levels of muscle activation when feedback was present. From these results, we can see that each participant exhibited different postural and muscle activation behavior, particularly in response to the presence of feedback.

### 3.2. Ergonomic Assessments

**Figure 4:**
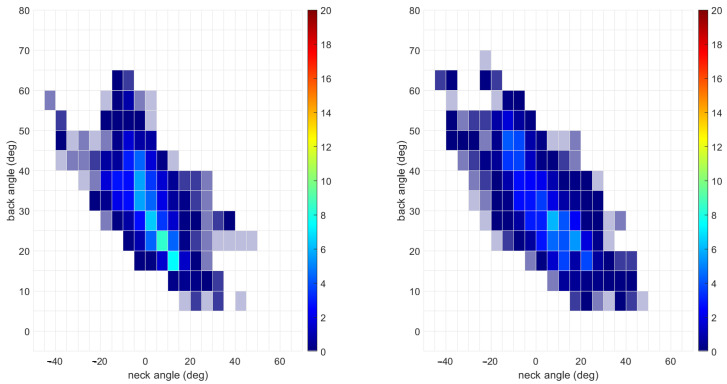
Heatmap for relative amount of time spent in each five-degree bin corresponding to different back and neck angle combinations. The plot on the left is averaged across trials without feedback, and the plot on the right is averaged across trials with feedback. The units for the color bars are the percentage of the total trial time.

**Figure A1:**
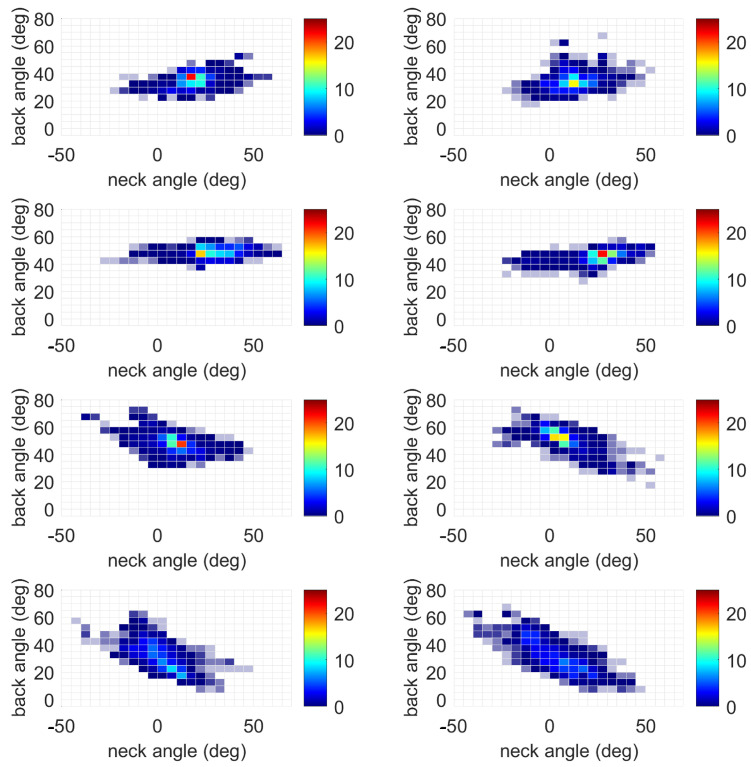
Heatmap for relative amount of time spent in each five-degree bin corresponding to different back and neck angle combinations. Each row is a participant. The first column is for trials without feedback, and the second column is for trials with feedback. The units for the color bars are the percentage of the total trial time.

Figure 4 shows heatmaps for the percent time spent in each bin averaged across participants when feedback was and was not present. The concentration of time spent in any one posture was generally reduced, indicated by colors closer to the blue end of the spectrum. Relative time heatmaps for each participant are included in Figure A1.

**Figure 5:**
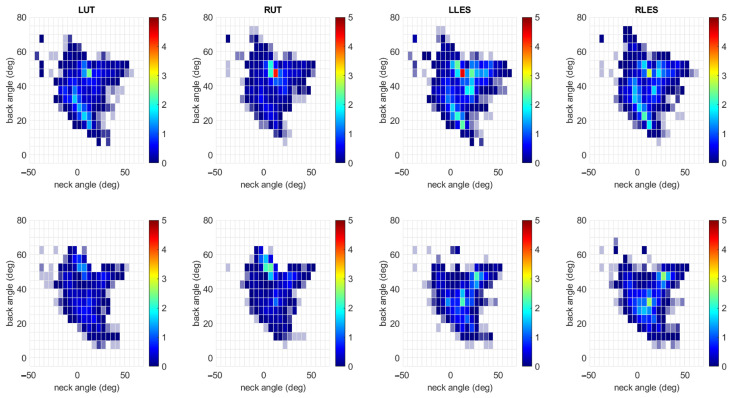
Heatmap for average iEMG for each muscle each back and neck angle combination bin. Each column is for a different muscle. The top (bottom) row is for trials without (with) feedback. The units of the color bar are the percent of the MVC multiplied by seconds.

Figure 5 shows heatmaps for the average iEMG for each muscle according to the back and neck angle bin when feedback was and was not present. These heatmaps are also averaged across participants. For most muscles, the iEMG in any one posture was reduced, indicated by colors closer to the blue end of the spectrum.

**Figure 6:**
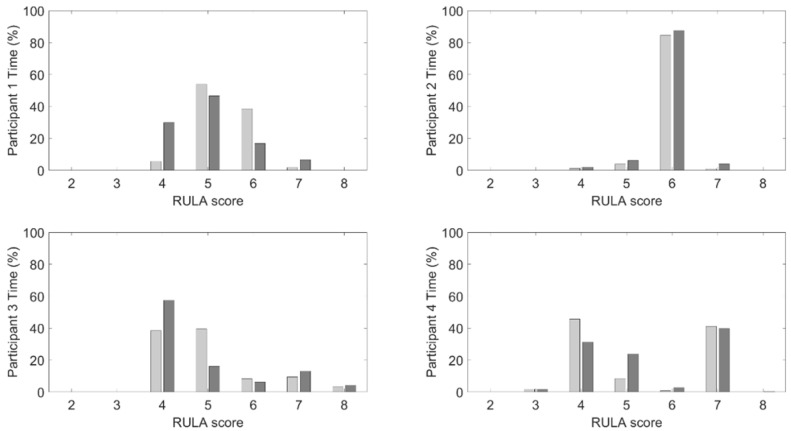
Relative times spent in each RULA bin when feedback was present (dark gray) and was not present (light gray).

For the results following, the data are presented for each participant to highlight the difference in the behaviors exhibited during the procedures without feedback present, indicating that each participant has a distinct approach to how they complete their work. It also has the added benefit of illustrating how each participant responds to the feedback. To translate the reported descriptive statistics to ergonomic risk, Figure 6 shows the relative amount of time spent in each RULA bin defined by the combination of neck and back angles for each participant with and without feedback. Table 2 reports each bin’s total amount of time weighted by the RULA score related to that bin.

**Figure 7:**
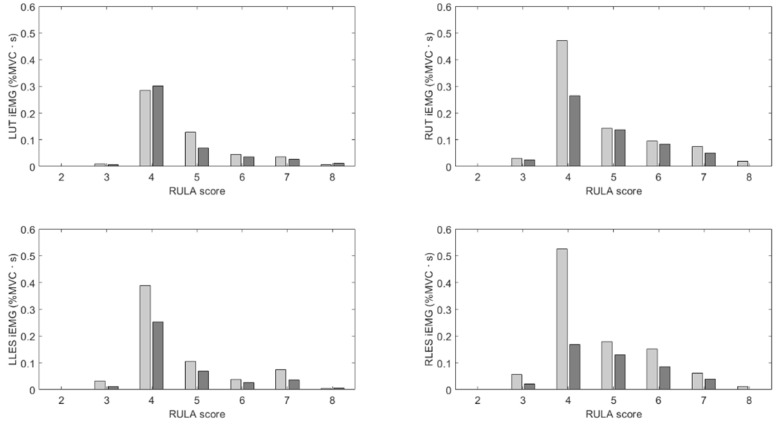
Relative times spent in each RULA bin when feedback was present (dark gray) and was not present (light gray).

Similarly, Figure 7 shows the iEMG for the amount of time spent in each RULA bin for each participant with and without feedback, and Table 3 reports the weighted averages. While there are no clear trends for the relative times, the muscle activation results show that the weighted averages were reduced when feedback was present.

These results suggest that participants adopted more varied postures and reduced the absolute magnitude of their muscle activation in response to feedback. However, the ways in which each participant alters their postures have different ergonomic risk implications associated with the new strategy. On the other hand, the postures were adjusted such that the weighted averages of iEMG were lower when feedback was provided, suggesting that all participants were able to reduce muscle activation in postures associated with higher ergonomic risk even if some participants spent more time in those postures.

### 3.3. Statistical Analysis

For the interested reader, Appendix A documents and organizes the ANOVA and post hoc Tukey results presented next in Table A1, Table A2, Table A3 and Table A4.

#### 3.3.1. Neck Angle

A significant main effect of activity (F(3, 1582) = 2.91, *p* = 0.03, $\eta^{2}$ = 0.01) indicates that neck angle differences were significant, but small. Post hoc comparisons showed that only Inspection and Air/Water Application were significantly different (*p* = 0.03, d = 0.02); the positive but small effect size implies that the neck angle was slightly greater during Inspection. A significant main effect for feedback was also found (F(1, 1582) = 33.91, *p* < 0.001, $\eta^{2}$ = 0.02). Post hoc comparisons show that feedback significantly affected neck angle (*p* < 0.001, d = −0.43). The negative, medium effect size implies that the neck angle was greater when feedback was present.

#### 3.3.2. Back Angle

A significant main effect of activity (F(3, 1582) = 4.07, *p* < 0.01, $\eta^{2}$ = 0.01) shows small significant differences in back angle. Post hoc comparisons using Tukey’s test indicated that only Drilling and Inspection were significantly different (*p* = 0.02, d = 0.02), with a small positive effect size implying that the back angle is slightly larger during Drilling than Inspection. A significant main effect of feedback was not found for back angle (F(1, 1582) = 1.22, *p* = 0.27, $\eta^{2}$ < 0.01).

#### 3.3.3. Left and Right Upper Trapezius Integrated EMG

A significant main effect of activity (F(3, 1699) = 59.46, *p* < 0.001, $\eta^{2}$ = 0.09) indicates medium to large significant differences in LUT iEMG. Similarly, a significant main effect of activity (F(3, 1699) = 51.33, *p* < 0.001, $\eta^{2}$ = 0.08) with medium to large significant differences in RUT iEMG was found. Post hoc comparisons indicated that Drilling was significantly different from Inspection (*p* < 0.001, d = 0.52), Drill Bit Switch (*p* < 0.01, d = 0.07), and Air/Water Application (*p* < 0.001, d = 0.19). The positive effect sizes mean that the LUT iEMG is greater during Drilling than the other activities. Post hoc comparisons for RUT iEMG showed similar results, namely that Drilling was different from Inspection (*p* < 0.001, d = 0.47), Drill Bit Switch (*p* < 0.01, d = 0.07), and Air/Water Application (*p* < 0.001, d = 0.22). A significant main effect of feedback was also found for LUT iEMG (F(1, 1699) = 107.37, *p* < 0.001, $\eta^{2}$ = 0.05) and RUT iEMG (F(1, 1699) = 22.98, *p* < 0.001, $\eta^{2}$ = 0.01). Post hoc comparisons indicated that feedback significantly affected LUT (*p* < 0.001, d = 0.42) and RUT (*p* < 0.001, d = 0.14), with positive effect sizes, meaning that the iEMG was lower when feedback was present.

#### 3.3.4. Left and Right Erector Spinae Integrated EMG

A significant main effect of activity was found for the LLES (F(3, 1699) = 6.91, *p* < 0.001, $\eta^{2}$ = 0.01) and RLES (F(3, 1699) = 12.34, *p* < 0.001, $\eta^{2}$ = 0.02), with small significant differences in iEMG depending on the activity in which the participants were engaged. Post hoc comparisons indicated that Drilling was significantly different from Inspection (*p* < 0.001, d = 0.09); the positive effect size implies that the LLES iEMG was greater during Drilling. For RLES, Drilling was also significantly different from Inspection (*p* < 0.001, d = 0.17) as well as Air/Water Application (*p* = 0.04, d = 0.01). The positive effect sizes imply that the RLES iEMG was greater during Drilling than the other two activities. While a significant main effect of feedback was not found for the LLES iEMG (F(1, 1699) = 3.40, *p* = 0.07, $\eta^{2}$ < 0.01), a significant main effect was found for the RLES iEMG (F(1, 1699) = 32.27, *p* < 0.001, $\eta^{2}$ = 0.02), though the effect size is small. Post hoc comparisons indicated that feedback significantly affected RLES (*p* < 0.001, d = −0.40), the negative effect size implying that the iEMG was higher when feedback was present.

#### 3.3.5. Summary

These results suggest that small but statistically significant differences in neck and back angles were found across activities. Feedback slightly increased the neck angle but did not significantly alter the back angle. Drilling was associated with higher muscle activation in the upper trapezius and erector spinae compared to other tasks. While feedback on average reduced muscle activation in the trapezius muscles, it caused a slight increase in muscle activation for the right erector spinae.

## 4. Discussion

This feasibility study explored the use of a novel wearable sensor-based vibrotactile feedback system to facilitate ergonomic training by promoting ergonomic awareness and training during a standard dental procedure. Interestingly, the most notable outcome of the posture-driven vibrotactile feedback was a reduction in muscle activation. The most consistent effect of the haptic feedback system was observed in a reduction in upper trapezius muscle activation on both sides, though these reductions occurred in the presence of a statistically significant increase in neck angles. However, it should be noted that the neck angles remained on average below the feedback threshold of 20 degrees without (9.7 degrees) and with feedback (13.9 degrees). These changes, however, are relevant given the established link between sustained neck flexion and the development of neck and shoulder pain in dental professionals [6]. The decrease in muscle activity when feedback was present suggests that participants were more aware of their muscle activation and adjusted their muscle recruitment accordingly, despite the slight increase in neck angle. This finding aligns with prior research on intraoperative micro-breaks in surgical settings, which have been shown to reduce fatigue and improve posture without compromising task performance [14,15]. Here, the reduced muscle strain is achieved by providing a vibrotactile stimulus reminding the user to regularly adopt a different posture.

Feedback did not significantly affect the back angle or left erector spinae activation. This finding may be due to the nature of the dental procedure, which requires forward trunk flexion that is difficult to avoid without additional ergonomic equipment such as a microscope or specialized prismatic loupes. Future studies could consider evaluating the effects of such equipment utilizing a wearable sensor system to better understand how the users adapt to the change in their environmental set up. Alternatively, the feedback thresholds or delivery may not have been sensitive enough to prompt consistent changes to improve back posture. Future work should consider different threshold levels or potentially adaptive thresholds that can respond to muscle fatigue. The right erector spinae activation did incur an increase in muscle activity, but the effect sizes suggested that the change was small. These findings highlight the importance of tailoring feedback systems to the specific biomechanical demands of dental tasks and suggest that future iterations may benefit from more nuanced or adaptive feedback algorithms [13]. It is possible that designing feedback to incorporate muscle activation data could also benefit the user. It is possible that future iterations of ergonomic equipment and assistive devices may be able to offload muscle strain during a procedure while a dentist is in a traditionally mechanically disadvantageous posture. For example, several research groups have explored the use of trunk exoskeletons to offload back muscle loading in a number of different applications including dentistry [40,41], surgery [42,43], and industrial tasks [44].

The results regarding the effects of activity type on posture and muscle activation were also notable. In particular, drilling was associated with higher back angles, greater upper trapezius activation, and greater lower erector spinae activation compared to other activities. This finding also reinforces the notion that task-specific ergonomic interventions may be required and provides additional evidence that certain procedures, or tasks within those procedures, may carry a higher risk of MSD development. Educators may consider incorporating targeted ergonomic training during these high-risk activities to reinforce healthy movement patterns. Quantifying the risk associated with different procedures may also be useful when scheduling appointments, with enough rest planned between high-risk activities for recovery [45].

The findings support the theory that biomechanical awareness, when prompted through real-time feedback, can lead to adaptive motor responses even in novice practitioners, suggesting that wearable feedback systems may serve as effective tools for embedding ergonomic principles into procedural memory during early clinical training. From an educational perspective, the integration of real-time feedback into preclinical training offers several advantages. First, it provides immediate, objective information about posture, which may be more impactful than retrospective ergonomic instruction [13]. Second, it encourages students to develop self-awareness and self-regulation skills that are essential for long-term musculoskeletal health. Finally, it aligns with competency-based education models by offering individualized, performance-based feedback that can be integrated into existing simulation-based curricula [46].

If a dental student reports or is being treated for an MSD, the authors of [45] recommend that the dental faculty assess the student’s understanding of ergonomics through extended observation during clinical and simulation activities. They also highlighted the potential to implement real-time feedback to correct harmful habits, and it is likely that the symptoms will recur post-treatment unless the root cause of the ergonomic issue is determined. Additionally, to address the ergonomic shortcomings in dental education, schools can take proactive steps to mitigate the inevitable development of poor ergonomic behaviors that arise when students are required to work alone, even though dentistry is most effectively practiced with an assistant. One effective strategy is to adapt the curriculum to ensure that students have access to peer support during procedures that demand suctioning, mirror clearing, and moisture control, particularly during times of drilling, in which ergonomic form is often compromised by the need to hold a suction device instead of a mirror. Integrating modern dental innovations such as Isovacs or combination devices that consolidate suction, air, water, and mirror functions can further support ergonomic practices, especially when paired with proper supervision and haptic feedback mechanisms. Early education, hands-on training, and timely correction are essential to reducing the incidence of musculoskeletal disorders (MSDs) among dental students and promoting long-term occupational health.

### Current Limitations

The small sample size and single-institution design limit generalizability. Although the statistical power for this study is approximately 10%, indicating limited sensitivity to detect small effects, the findings still offer valuable preliminary insights to inform future research directions, help refine hypotheses, and contribute to the broader understanding of the topic. While the results should be interpreted with caution, the study lays important groundwork for more robust future investigations. Additionally, the use of manikins rather than live patients may not fully replicate the physical and cognitive demands of clinical care. With respect to the sEMG data, a potential uncontrolled error may have been introduced by having a research team member provide the opposing force during the MVC exercises instead of a dynamometer. Additionally, it is possible that skin motion artifacts (i.e., relative motion between the IMU and the underling skeletal structure) may have affected the accuracy of the spinal orientation estimates.

Despite these limitations, this study provides valuable preliminary evidence supporting the feasibility and potential benefits of wearable feedback systems in dental education. Future research should explore the long-term effects of feedback on posture retention, the integration of other feedback modes (e.g., visual or auditory cues), and the impact of such systems on clinical performance and patient outcomes. Future work should explore adaptive thresholds based on posture and possibly muscle activation variability to better tailor feedback timing to individual users and task demands. Larger, multi-institutional, longitudinal studies are needed to validate these findings and inform best practices for implementation.

## 5. Conclusions

This feasibility study demonstrates that a wearable sensor-based vibrotactile feedback system can influence posture and muscle activation patterns in dental students during a standard procedure. While feedback led to increased neck flexion, it reduced upper trapezius muscle activation, suggesting a shift in muscle recruitment that may reduce fatigue. The system did not significantly alter back posture or left erector spinae activity, highlighting the need for task-specific feedback strategies and magnification wear that allows for ideal neck and back posture at rest that does not compromise vision of the surgical field. These findings support the integration of real-time ergonomic feedback into dental education to promote healthier biomechanical behaviors and potentially mitigate the risk of MSDs. Future research should explore long-term retention, adaptive feedback mechanisms, and broader implementation across diverse clinical settings.

## Figures and Tables

**Table 1:** Average procedure time, neck angle, and back angle without and with feedback by participant. Note that the standard deviations are reported for the neck and back angles.

Participant	Without Feedback			With Feedback		
	Time (min)	Neck Angle (deg)	Back Angle (deg)	Time (min)	Neck Angle (deg)	Back Angle (deg)
1	18.1	18.9 (7.4)	35.2 (4.3)	25.4	12.3 (7.9)	34.2 (5.0)
2	16.6	30.3 (9.4)	48.4 (3.1)	14.8	26.6 (10.1)	45.9 (3.3)
3	15.5	8.8 (9.5)	48.0 (5.5)	15.1	7.4 (8.4)	51.0 (6.1)
4	22.4	1.4 (9.0)	31.2 (8.7)	16.0	3.7 (11.8)	31.2 (10.6)

**Table 2:** Weighted averages for each participant’s relative times spent in RULA scores.

	Participant 1	Participant 2	Participant 3	Participant 4
No Feedback	0.67	0.67	0.62	0.66
With Feedback	0.62	0.74	0.59	0.68

**Table 3:** Weighted averages for each participant’s relative times spent in RULA scores.

	LUT	RUT	LLES	RLES
No Feedback	0.298	0.493	0.370	0.577
With Feedback	0.258	0.336	0.234	0.272

## Data Availability

The de-identified datasets may be available upon reasonable request.

## Appendix A

Figure A1 shows heatmaps for the percent time spent in each bin for each participant when feedback was and was not present. The concentration of time spent in any one posture was generally reduced, indicated by colors closer to the blue end of the spectrum.

These tables document the statistical results reported in the main body of the paper, with Table A1 reporting the main effects of feedback and Table A2 reporting the main effects of activity. As a reminder, the muscles include the left and right upper trapezius (LUT and RUT, respectively) as well as the left and right lower erector spinae (LLES and RLES, respectively).

**Table A1:** ANOVA results for main effects of feedback as detailed in Section 3.3, including the F-statistic, degrees of freedom (DOF), *p*-value, and effect size ($\eta^{2}$).

Variable	F-Statistic	DOF	*p*-Value	$\eta^{2}$
Neck Angle	33.91	1, 1582	<0.001	0.02
Back Angle	1.22	1, 1582	0.27	<0.01
LUT	107.37	1, 1699	<0.001	0.05
RUT	22.98	1, 1699	<0.001	0.01
LLES	3.40	1, 1699	0.07	<0.01
RLES	32.27	1, 1699	<0.001	0.02

**Table A2:** ANOVA results for main effects of activity as detailed in Section 3.3, including the F-statistic, degrees of freedom (DOF), *p*-value, and effect size ($\eta^{2}$).

Variable	F-Statistic	DOF	*p*-Value	$\eta^{2}$
Neck Angle	2.91	3, 1582	0.03	0.01
Back Angle	4.07	3, 1582	<0.01	0.01
LUT	59.46	3, 1699	<0.001	0.09
RUT	51.33	3, 1699	<0.001	0.08
LLES	6.91	3, 1699	<0.001	0.01
RLES	12.34	3, 1699	<0.001	0.02

The tables below document the post hoc Tukey analyses for significant ANOVA results. For the feedback analyses in Table A3, the two groups being compared are no feedback and feedback. For the activity analyses in Table A4, the four groups being compared are Drilling, Inspection, Air/Water Application, and Drill Bit Switch. Only statistically significant results (i.e., p<α=0.05) are reported.

**Table A3:** Post hoc Tukey results for the main effect of feedback as detailed in Section 3.3, including the variable, *p*-value, and effect size (Cohen’s *d*).

Variable	*p*-Value	Cohen’s *d*
Neck Angle	<0.001	−0.43
LUT	<0.001	0.42
RUT	<0.001	0.14
RLES	<0.001	−0.40

**Table A4:** Post hoc Tukey results for the main effect of activity as detailed in Section 3.3, including the variable, the groups being compared, the *p*-value, and the effect size (Cohen’s *d*).

Variable	Group 1	Group 2	*p*-Value	Cohen’s *d*
Neck Angle	Inspection	Application	0.03	0.02
Back Angle	Drilling	Inspection	0.02	0.02
LUT	Drilling	Inspection	<0.001	0.52
LUT	Drilling	Drill Bit Switch	<0.01	0.07
LUT	Drilling	Application	<0.001	0.19
RUT	Drilling	Inspection	<0.001	0.47
RUT	Drilling	Drill Bit Switch	<0.01	0.07
RUT	Drilling	Application	<0.001	0.22
LLES	Drilling	Inspection	<0.001	0.09
RLES	Drilling	Inspection	<0.001	0.17
RLES	Drilling	Application	0.04	0.01
